# On the Mortality from Phthisis in Prisons

**Published:** 1848-05

**Authors:** 


*On the mortality from phthisis in Prisons.*—Among the prisoners in the Pentonville Prison, phthisis has been considerably reduced by attention to those suspicious circumstances which were likely to excite the disease. From the opening of the prison to the termination of 1844, the annual mortality per 1000 from phthisis had amounted to 11.47. The physician, Dr. G. O. Rees, suspected that the *dusty trades* carried on in the cells, might have added to the chances of death by this disease. In 1845, measures were taken to guard against the supposed causes: in 1846 only four cases per 1000 of consumption occurred, and in 1847, up to the 20th of October, there was not a single death from the disease.

The annual mortality of the Pentonville prisoners is 15.7 per 1000,—that of the Foot-guards 21.6: and it is remarkable that the mortality from consumption alone in the Guards is nearly as high (14.1 per 1000) as the total mortality of Pentonville prisoners,—men who are about the same age, on an average, as the soldiers.—*Ibid.*

